# The effect of noise exposure on insulin sensitivity in mice may be mediated by the JNK/IRS1 pathway

**DOI:** 10.1186/s12199-018-0694-3

**Published:** 2018-02-12

**Authors:** Lijie Liu, Cong Fang, Jing Yang, Hongyu Zhang, Yi Huang, Chuanying Xuan, Yongfang Wang, Shengwei Li, Jun Sha, Mingming Zha, Min Guo

**Affiliations:** 1 Medical College, Southeast University, No.87, Dingjiaqiao Street, Gulou, Nanjing, China; 2 Institute of Life Sciences, Southeast University, Nanjing, China

**Keywords:** Noise exposure, Insulin sensitivity, JNK/IRS1 pathway, Inflammation, Oxidative stress

## Abstract

**Background:**

Epidemiological studies have suggested that noise exposure may increase the risk of type 2 diabetes mellitus (T2DM), and experimental studies have demonstrated that noise exposure can induce insulin resistance in rodents. The aim of the present study was to explore noise-induced processes underlying impaired insulin sensitivity in mice.

**Methods:**

Male ICR mice were randomly divided into four groups: a control group without noise exposure and three noise groups exposed to white noise at a 95-dB sound pressure level for 4 h/day for 1, 10, or 20 days (N1D, N10D, and N20D, respectively). Systemic insulin sensitivity was evaluated at 1 day, 1 week, and 1 month post-noise exposure (1DPN, 1WPN, and 1MPN) via insulin tolerance tests (ITTs). Several insulin-related processes, including the phosphorylation of Akt, IRS1, and JNK in the animals’ skeletal muscles, were examined using standard immunoblots. Biomarkers of inflammation (circulating levels of TNF-α and IL-6) and oxidative stress (SOD and CAT activities and MDA levels in skeletal muscles) were measured via chemical analyses.

**Results:**

The data obtained in this study showed the following: (1) The impairment of systemic insulin sensitivity was transient in the N1D group but prolonged in the N10D and N20D groups. (2) Noise exposure led to enhanced JNK phosphorylation and IRS1 serine phosphorylation as well as reduced Akt phosphorylation in skeletal muscles in response to exogenous insulin stimulation. (3) Plasma levels of TNF-α and IL-6, CAT activity, and MDA concentrations in skeletal muscles were elevated after 20 days of noise exposure.

**Conclusions:**

Impaired insulin sensitivity in noise-exposed mice might be mediated by an enhancement of the JNK/IRS1 pathway. Inflammation and oxidative stress might contribute to insulin resistance after chronic noise exposure.

## Background

Noise, one of the most widespread sources of environmental pollution, is considered not only an environmental nuisance but also a threat to public health [1]. Beyond the well-recognized problem of hearing impairment, increasing attention is being paid to the cumulative adverse effects of noise exposure on extra-auditory systems [1]. In a more than 10-year prospective epidemiological study conducted by Sorensen et al. in a Danish cohort, each 10-dB increase in average road traffic noise at the current residence was found to be associated with a statistically significant 11% increased risk of incident diabetes; this risk increased to 14% when road traffic noise was estimated for all the places in which an individual had lived during the previous 5 years [2]. Their study raised concern about noise as a risk factor of diabetes, particularly given the global epidemic of this disease and the increasingly widespread pattern of noise pollution [2, 3]. A previous study by our group indicated that noise exposure at a 95-dB sound pressure level (dB SPL) induces insulin resistance in mice [4]. More recently, another experimental study reported abnormalities in glucose regulation and insulin sensitivity in rats that were chronically exposed to noise at a 100-dB SPL [5]. Because insulin resistance is well known to be a key contributor to type 2 diabetes mellitus (T2DM) and a key pathological feature of T2DM, these experimental studies together suggested a contributory role of noise exposure to increasing the risk of T2DM. Considering the alarming global epidemic of T2DM [6] and the global prevalence of noise pollution [1], it is imperative to explore the mechanisms underlying the impairment of insulin sensitivity after noise exposure.

We previously examined circulatory corticosterone levels in mice subjected to noise exposure at a 95-dB SPL at 4 h/day for 1, 10, and 20 days. The collected data showed that at 1 day after the cessation of noise exposure, the levels of plasma corticosterone in all three noise groups were significantly increased compared to those in matched control groups [4]. This result was consistent with other reports indicating that noise is a source of environmental stress [7–9]. Furthermore, these data illustrated a sustained stress response to chronic noise and support the notion that the animals did not adapt to the noise, even after prolonged exposure [10].

Persistently elevated cortisol levels have been proposed to be associated with the development of insulin resistance [11]. Positive associations between excess stress hormones, oxidative stress, and insulin resistance have been widely reported [11–13]. It is also well documented that the release of inflammatory cytokines, such as tumor necrosis factor-α (TNF-α) and interleukin-6 (IL-6), which are often cited as crucial cytokines that mediate insulin resistance [14, 15], can be induced by chronic stress [16, 17]. The protein c-Jun N-terminal kinase (JNK) has been increasingly recognized as an important mediator of insulin resistance that is associated with inflammation and oxidative stress [18] through the phosphorylation of serine residues in insulin receptor substrate-1 (IRS-1) [15]. Considering these facts, we postulated that the adverse effects of noise exposure on insulin sensitivity might be mediated, at least in part, through complex interactions between inflammation, oxidative stress, and the JNK/IRS1 pathway. Thus, in the present study, we evaluated the influence of noise exposure (95 dB SPL, 4 h/day for 1, 10, or 20 consecutive days) on systemic insulin sensitivity, the JNK/IRS1/Akt pathway, and markers of inflammation and oxidative stress in ICR mice.

## Methods

### Animals and experimental protocol

**Fig. 1:**
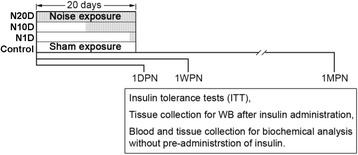
Experimental timeline. The animals were randomly divided into four groups: a control group without noise exposure and three noise groups exposed to white noise at a 95-dB SPL at 4 h/day for 1, 10, or 20 days (N1D, N10D, and N20D, respectively) as indicated by the gray area within the 20-day period. The animals in each group were further subdivided into three subgroups according to when end-point evaluations were performed [1 day, 1 week, and 1 month post the cessation of noise exposure (1DPN, 1WPN, and 1MPN, respectively)]

Five-week-old wild-type male ICR mice were obtained from the Qinglongshan Animal Center (Nanjing, China, SCXK(SU)2012-0008). In total, 144 mice were used in this study. The mice were housed in conventional cages with a 12-h light/dark cycle (lights on at 7 am) and had free access to food (standard rodent chow, SHOOBREE, Xietong Organism, Jiangsu, China) and water. After a 1-week acclimation period, the animals were randomly assigned into one control group or into one of three noise-exposure groups. The animals in the noise-exposure groups were exposed to broadband noise at a 95-dB SPL for 4 h/day between 8:00 am and 12:00 pm for 1 day (the N1D group), 10 days (the N10D group), or 20 days (the N20D group). The animals in each of the noise-exposure groups were further subdivided into three subgroups according to the times at which the assessments were performed: at 1 day, 1 week, or 1 month after the final noise exposure (1DPN, 1WPN, and 1MPN, respectively) (Fig. 1). The mice in the control group were also subdivided into the same subgroups according to the assessment time points and served as age-matched controls. The animals were treated humanely and with regard for the alleviation of suffering. Food consumption per cage was measured every 3 or 4 days by subtracting the amount of the food left from the initial amount of food supplied. To avoid circadian rhythm-induced variations, the insulin tolerance test (ITT) was always initiated at 9:00 am, and tissue and blood samples used for further studies were collected between 2:00 pm and 3:00 pm. All of the animal procedures were approved by the University Committee for Laboratory Animals of Southeast University, China (reference number: 20130307-004).

### Noise exposure

The animals were exposed to noise as described previously [4]. Briefly, after acclimatization to the noise-exposure setting for 30 min, awake and unrestrained mice were placed separately into metal net cages that were 50 cm below the horns of two loudspeakers. Electrical Gaussian noise generated by a System III processor from Tucker-Davis-Technologies (TDT, Alachua, FL, USA) was delivered to speakers after power amplification. In consistent with our previous studies, the acoustic spectrum of the sound was distributed mainly between 1 and 20 kHz [4, 19]. The noise level was monitored using a 1/4-in. microphone linked to a sound level meter (Larson Davis 824, Depew, NY, USA), and the sound intensity was maintained at a 95 ± 1 dB SPL.

### Insulin tolerance test

The insulin tolerance test (ITT) on 4-h-fasted mice was initiated at 9:00 am to avoid circadian rhythm-induced variation. Blood samples were obtained from the tail vein of awake mice just prior to (0 min) and at 30, 60, and 90 min after an intraperitoneal injection of insulin (Humulin, 0.75 U/kg body wt). Blood glucose levels were measured using a portable glucose monitor (Bayer Contour, Bayer HealthCare LLC, Whippany, NJ) and test strips. The time course of absolute blood glucose recorded during the ITT and the areas under the blood glucose curves (AUC) were used to evaluate insulin sensitivity. After completion of the test, the mice were returned to their home cage and given free access to food and water.

### Western blot analysis

Three to 4 h after completion of the ITT (i.e., 2:00–3:00 pm), the mice were decapitated 20 min after an intraperitoneal injection of insulin (Humulin, 0.75 U/kg body wt), and their gastrocnemius muscles were dissected and homogenized in ice-cold RIPA buffer (Beyotime P0013C, China) supplemented with a complete protease inhibitor cocktail (Roche, Germany) and PhosSTOP (Roche, Germany). The protein extracts (40 μg) for each preparation were separated using 10% SDS-PAGE and electrotransferred onto PVDF membranes (Millipore, Bedford, MA, USA). After blocking with Tris-buffered saline, 0.1% Tween 20, and 5% nonfat dry milk, the membranes were incubated with primary antibodies overnight at 4 °C. The following antibodies were used: anti-IRS1 (Cell Signaling Technology, Cat no. #2382, Beverly, MA, USA), anti-phospho-IRS1 (Ser307) (Cell Signaling Technology, Cat no. #2381, Beverly, MA, USA), anti-JNK (Cell Signaling Technology, Cat no. #9252, Beverly, MA, USA), anti-phospho-JNK (Thr183/Tyr185) (Cell Signaling Technology, Cat no. #9251, Beverly, MA, USA), anti-Akt (Cell Signaling Technology, Cat no. #4685, Beverly, MA, USA), anti-phospho-Akt (Ser473) (Cell Signaling Technology, Cat no. #4058, Beverly, MA, USA), and anti-phospho-Akt (Thr308) (Cell Signaling Technology, Cat no. #4056). The protein bands were visualized using an ECL Kit (WBKLS0050; Millipore, Billerica, MA, USA), and a densitometry analysis was performed using ImageJ.

### Biochemical analysis

Because insulin can influence the production of inflammatory cytokines and the oxidative stress response [20], separate groups of mice were used in the biochemical analyses. Blood was collected immediately from the trunk into dry tubes after the mice were decapitated without the pre-administration of insulin. Gastrocnemius muscles were then harvested, weighed, and homogenized to evaluate oxidative stress according to the protocols provided with assay kits for superoxide dismutase (SOD, a powerful endogenous enzymatic antioxidant) (STA-340, Cell Biolabs, Inc.), catalase (CAT, an important endogenous enzymatic antioxidant) (Cell Biolabs, STA-341, USA), and malondialdehyde (MDA, a major secondary oxidation product) (Cell Biolabs, STA-832, USA). Following centrifugation at 4 °C, plasma was separated from each sample, and plasma concentrations of TNF-a and IL-6 (Cat#EMC102a and Cat#EMC004, NeoBioscience, Shenzhen, China) were determined using enzyme-linked immunosorbent assay kits according to the manufacturer’s instructions and guidelines.

### Statistical analysis

The data are expressed as means ± standard errors (SE). Depending on the type of measurement, two-way or one-way ANOVAs were performed with a focus on the effect of noise exposure (grouping). Post hoc pairwise comparisons between each noise group and the control group were performed (Tukey’s method) if a significant effect of noise exposure was detected. Significance was assumed at *p* < 0.05.

## Results

### Effect of noise exposure on systemic insulin sensitivity in mice

**Fig. 2:**
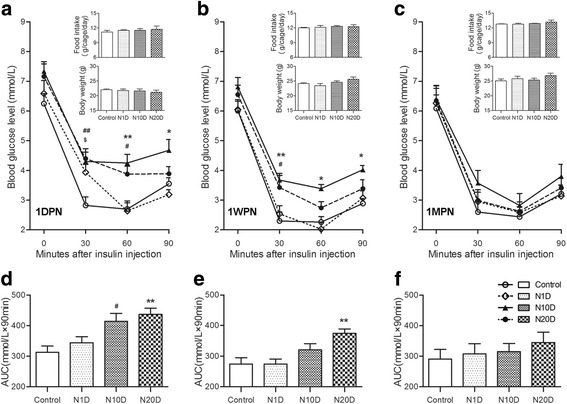
Effect of noise exposure on systemic insulin sensitivity in mice. **a–c** Blood glucose levels during the ITTs in 4-h-fasted mice were assessed at 1DPN, 1WPN, and 1MPN. The inserts show the average daily food intake of the corresponding animals measured on a per-cage basis and body weight measured just before the ITT. **d**–**f** The areas under the curves (AUC) for blood glucose are shown in **a**–**c**, respectively. Values are presented as the means ± SEM of 8 mice per group. *p < 0.05 and **p < 0.01 indicate significance in post hoc comparisons between N20D and the control group after a two-way RM (**a**–**c**) or one-way (**d**–**f**) ANOVA, showing a significant effect of noise. ^#^*p* < 0.05 and ^##^*p* < 0.01: N10D vs control group. ^$^*p* < 0.05: N1D vs control group

The food intake and the bodyweight of all groups tested at the three time points were similar (Fig. 2, inserts). At 1DPN, all noise groups exhibited blunted glucose responses to insulin challenge, as indicated by significantly higher blood glucose level(s) at one or more time point(s) during the ITT and larger AUC values (which were significant for N10D and N20D) compared with the control values (Fig. 2a, d). At 1WPN, the N1D group showed similar insulin sensitivity to that of the control group, and the N10D group only exhibited a significantly high blood glucose level at 30 min after insulin injections. However, the N20D group displayed not only significantly higher glucose levels at all three test points after insulin injections but also a significantly higher value of AUC (Fig. 2b, e). No differences were observed between any of the groups at 1MPN (Fig. 2c, f).

### Effect of noise exposure on the JNK/IRS1/Akt pathway in the gastrocnemius muscle

**Fig. 3:**
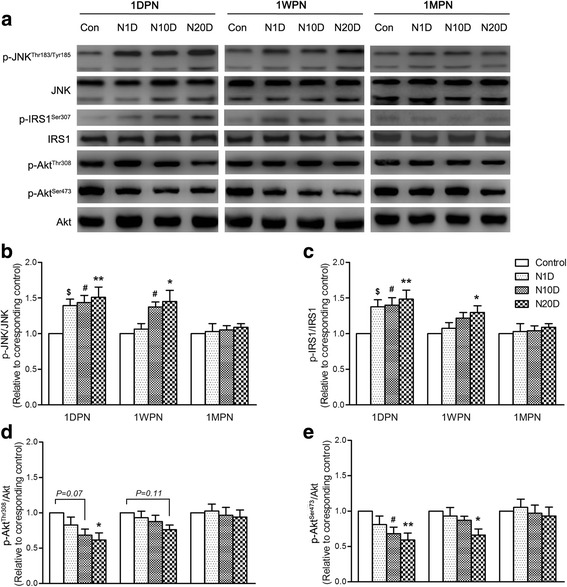
Effect of noise exposure on insulin signaling pathways in the gastrocnemius muscle. **a** Levels of total and Thr^183^/Tyr^185^ phosphorylated JNK, total and Ser^307^ phosphorylated IRS1, and total Akt and phosphorylated Akt at Ser^473^ and Thr^308^ were detected via immunoblotting, and representative Western blots are presented. **b**–**e** Graphs showing the relative intensities of phospho-protein bands are normalized to the corresponding total protein levels in each case. The data are expressed relative to values for age-matched controls. The average of each age-matched control group was set to 1. Values are presented as the mean ± SEM of 8 mice per group. *p < 0.05 and **p < 0.01 indicate significantly different mean values in the post hoc comparisons between N20D and the control after a one-way ANOVA, demonstrating a significant effect of noise. ^#^*p* < 0.05 indicates N10D vs control group. ^$^*p* < 0.05 indicates N1D vs control

As demonstrated in Fig. 3, at 1DPN, the phosphorylation of JNK at Thr^183^/Thr^185^ and IRS1 at Ser^307^ were significantly elevated in all three noise-exposed groups, while the Akt phosphorylation at Thr^308^ and Ser^473^ induced by exogenous insulin stimulation exhibited decreases in all three noise-exposed groups, which reached significance in the N20D group for Thr^308^ phosphorylation and in the N10D and N20D groups for Ser^473^ phosphorylation. At 1WPN, significantly elevated phosphorylation of JNK was observed in the N10D and N20D groups, while the significantly elevated IRS1 phosphorylation and blunted Akt phosphorylation were only shown in the N20D group. No significant difference in phosphorylation levels from the controls was identified in any of the noise-exposed groups at 1MPN.

### Effect of noise exposure on the levels of inflammatory and oxidative stress markers

To explore whether inflammation and/or oxidative stress might be involved in the influence of noise exposure on insulin sensitivity, we examined the circulating levels of inflammatory cytokines (TNF-α and IL-6) and the tissue levels of oxidative stress markers (SOD and CAT activities and MDA concentrations) in mice subjected to 1 day and 20 days of noise exposure. Since no significant difference in the ITT and Western blotting assay was revealed between the groups at 1MPN, we did not perform these biochemical assays at 1MPN.

**Fig. 4:**
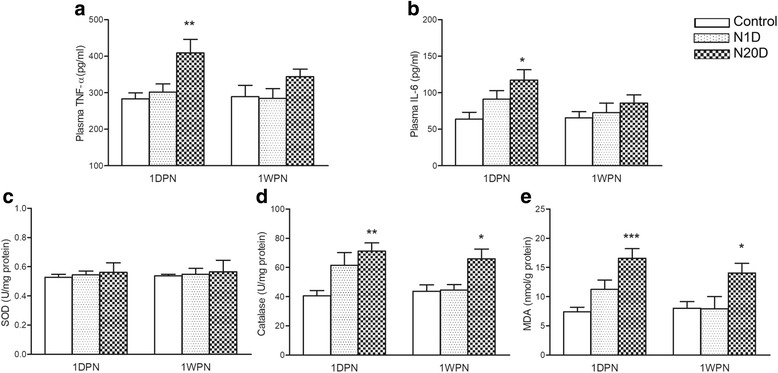
Effect of noise exposure on biomarkers of inflammation and oxidative stress. **a**, **b** Plasma levels of TNF-α (**a**) and IL-6 (**b**). **c**–**e** Activity of SOD (**c**) and CAT (**d**) and the level of MDA (**e**) in the gastrocnemius muscle. Values are presented as the mean ± SEM of 8 mice per group. *p < 0.05, **p < 0.01, and ***p < 0.001 indicate significantly different mean values in post hoc comparisons between N20D and the control after a one-way ANOVA

As illustrated by Fig. 4, the levels of the tested cytokines and oxidative stress markers were all comparable between the control and N1D groups at both 1DPN and 1WPN. However, the N20D group exhibited significantly higher plasma TNF-α and IL-6 levels at 1DPN and significantly elevated CAT activity and MDA concentrations in their skeletal muscles at both 1DPN and 1WPN, suggesting a transient elevation in systemic inflammatory responses and a prolonged oxidative imbalance in the skeletal muscle of the animals that were chronically subjected to repeated noise.

## Discussion

As in our previous work, the animals in this study were exposed to broadband noises presented at 95 dB for 4 h/day, which is equivalent to 90 dBA for 8 h (dBA is the sound pressure level when an “A” contour filter is used according to the sensitivity of the human ear). The recommended exposure limit in workplaces according to the Occupational Safety and Health Administration (OSHA) is 90 dBA for 8 h. Therefore, the exposure setting used in this study is not higher than the occupational safety allowance. Similar to our previous observations, the blunted glucose response observed during the ITT was shown in all noise-exposed groups at 1DPN and was also evident at 1WPN in the N10D and N20D groups in this study, indicating a trend towards insulin resistance with prolonged noise exposure.

Skeletal muscles are the most important insulin-targeted tissue involved in maintaining whole-body glucose homeostasis under insulin-stimulated conditions and are major sites of insulin resistance in T2DM subjects. In skeletal muscle, insulin binds to a surface receptor and triggers a cascade of signaling events involving IRS-1 and Akt that induces the translocation of the glucose transporter from its intracellular depot(s) to the cell surface, where these transport proteins mediate the uptake of glucose into the cell. Defects in these signaling pathways are considered the major pathogenic disturbances underlying the development and progression of insulin resistance [21]. Decreased Akt phosphorylation at the insulin-responsive active site (Ser^473^ and Thr^308^) following insulin stimulation have been well documented in insulin resistance [22]. In the present study, noise-exposed animals exhibited a blunted glucose response to insulin injection, which was accompanied by decreased Akt phosphorylation in the skeletal muscle, indicating an impairment of skeletal muscle insulin sensitivity.

Noise has long been classified as an environmental stress. Several studies (including the two studies illustrating the development of insulin resistance in animals subjected to chronic noise) have reported significant increases in plasma stress hormone levels during and after various noises [4, 5, 10]. In our previous study, increased plasma corticosterone levels were observed at 1 day after noise exposure for 1, 10, and 20 days, suggesting a sustained stress response throughout the noise exposure in the N20D group [4]. Persistently elevated cortisol levels have been proposed to be an etiological factor of insulin resistance [11, 13, 23]. Therefore, we have proposed that stress responses might contribute to the development of insulin resistance in noise-exposed animals [4]. c-Jun N-terminal kinase (JNK) is an evolutionarily conserved stress-activated protein kinase (SAPK) that is activated primarily by inflammatory cytokines and exposure to environmental stress [24]. Recent studies have identified JNK as a crucial link between environmental challenges and metabolic regulation [25]. Activated JNK can phosphorylate IRS-1 at the inhibitory site Ser^307^ and therefore suppress insulin signal transduction [24]. The results of the present study indicated that systemic insulin resistance in the noise-exposed groups was accompanied by an increased activation of JNK with a corresponding increase in IRS-1 Ser^307^ phosphorylation in skeletal muscle tissue. Although our data do not allow us to rule out the contribution of other cellular serine kinases to the altered phosphorylation state of muscle IRS-1, it is highly plausible that JNK might serve as a candidate for the link between noise exposure and insulin resistance.

Accumulating evidence supports the notion that inflammatory markers can be induced after various stresses [16]. Inflammatory cytokines, including TNF-α and IL-6, are thought to contribute to the development of insulin resistance through the activation of several stress kinases, such as JNK [15]. As noise has long been realized as an environmental stress, we wondered whether inflammation might be involved in the adverse effect of noise exposure on insulin sensitivity. The data collected in this study show that the plasma concentrations of both TNF-α and IL-6 were comparable between the N1D and control group at both 1DPN and 1WPN, indicating that no inflammatory response was caused by the 1-day noise exposure. These inflammatory cytokines were significantly increased in the N20D group at 1DPN but not at 1WPN, suggesting that a temporary inflammatory response occurred in the animals that were subjected to chronic noise exposure. The recovery of inflammatory cytokine levels occurred earlier than that of insulin sensitivity, indicated by the same-as-control blood concentrations of TNF-α and IL-6 and the significantly blunted glucose response during ITT at 1WPN. The time courses of the inflammatory response, JNK activation, and the impaired insulin sensitivity in the N20D group suggest that the inflammation might be an event that links noise exposure with insulin resistance rather than a consequence of abnormal insulin sensitivity. Thus, consistent with the findings of Cui et al. in rat [5], our results provide more evidence that inflammation might contribute to the increased diabetes risk after chronic noise exposure.

Oxidative stress, defined as a disturbance in the balance between the production of reactive oxygen species and antioxidant defenses, has been proposed as a contributor to both the onset and the progression of insulin resistance [26]. As one of the major secondary oxidation products, MDA level has been regarded as reflecting the level of tissue damage caused by oxidative stress [27]. In the present work, the N20D group exhibited significant increases in MDA levels in skeletal muscles at both 1DPN and 1WPN, indicating enhanced oxidative stress. SOD and CAT are powerful endogenous enzymatic antioxidants that are responsible for protecting cells from oxidative damage [28]. In the present work, no differences in SOD activity were shown, whereas significant increases in CAT activity in skeletal muscle were exhibited in the N20D group at both 1DPN and 1WPN, suggesting an insufficient compensatory response to oxidative stress. Although the cross-sectional design of the present study does not permit us to determine the exact relationship between the insulin resistance, inflammatory cytokine levels and oxidative stress exhibited in the N20D group at both 1DPN and 1WPN, these data provide some information regarding the possible molecular mechanism underlying the non-auditory effects of noise pollution.

## Conclusions

We observed that insulin sensitivity impairment in noise-exposed animals was accompanied by an increase in JNK activation with a corresponding increase in IRS-1 serine phosphorylation in skeletal muscle tissue. Consistent with the possible causal role of inflammation and oxidative stress in JNK activation, we observed that the levels of circulatory inflammatory cytokines and oxidative stress markers in skeletal muscle were increased in animals that were exposed to noise for 20 days. These results suggest that the JNK/IRS pathway might mediate the adverse effect of noise exposure on skeletal muscle insulin sensitivity and that inflammatory response and oxidative stress are involved in the onset and/or development of insulin resistance after chronic noise exposure. Additional and more comprehensive methods (e.g., inhibiting JNK activity using JNK inhibitors) are required to reveal the exact relationships between these factors and their individual contributions to insulin resistance after noise exposure. The influence of noise exposure on the insulin sensitivity of liver and adipose tissue, the other two main types of insulin-sensitive tissues, should also be further investigated in future studies.

## Abbreviations

1DPN: 1-day post-noise exposure
1MPN: 1-month post-noise exposure
1WPN: 1-week post-noise exposure
Akt: Protein kinase B
AUC: Area under the curve
CAT: Catalase
dB SPL: Decibel sound pressure level
IL-6: Interleukin-6
IRS-1: Insulin receptor substrate-1
ITT: Insulin tolerance test
JNK: c-Jun N-terminal kinase
MDA: Malondialdehyde
N10D: Noise exposure for 10 days
N1D: Noise exposure for 1 day
N20D: Noise exposure for 20 days
SOD: Superoxide dismutase
TNF-α: Tumor necrosis factor-α
